# Pegvaliase Treatment for Adolescents With Phenylketonuria: A Multi‐Site Study

**DOI:** 10.1002/jmd2.70070

**Published:** 2026-02-17

**Authors:** Suzanne Hollander, Briana Valli, Erika Vucko, Melissa Lah, Amarilis Sanchez‐Valle, Brittany M. Murray, Amy Kritzer, Stephanie Sacharow

**Affiliations:** ^1^ Division of Genetics and Genomics Boston Children's Hospital Boston Massachusetts USA; ^2^ Department of Clinical Nutrition Boston Children's Hospital Boston Massachusetts USA; ^3^ Dr. Kiran C Patel College of Allopathic Medicine Nova Southeastern University Fort Lauderdale Florida USA; ^4^ Edwards Family Division of Genetics and Rare Diseases Lurie Children's Hospital Chicago Illinois USA; ^5^ Department of Medical and Molecular Genetics Indiana University School of Medicine Indianapolis Indiana USA; ^6^ Department of Pediatrics, Division of Genetics and Metabolism University of South Florida Health Tampa Florida USA; ^7^ Department of Pediatrics Harvard Medical School Boston Massachusetts USA

**Keywords:** adherence, adolescents, pegvaliase, phenylalanine, phenylalanine ammonia‐lyase, phenylketonuria

## Abstract

Phenylketonuria (PKU) is an inherited metabolic disorder causing elevated phenylalanine (Phe) levels and neurocognitive impairment if left untreated. While dietary therapy remains the treatment standard, adherence declines during adolescence. Pegvaliase, an injectable enzyme therapy approved for adults > 18 years in the United States, lowers Phe levels while allowing dietary flexibility. This study examines pegvaliase use in adolescents, focusing on efficacy, discontinuation patterns, and predictors of success. We conducted a retrospective chart review from four metabolic centers with 53 individuals with PKU (age 14–22 years) who initiated pegvaliase through March 2025. Data included demographics, treatment response, side effects, and discontinuation reasons. Mean pretreatment Phe was 716 μmol/L, which decreased by 44% post‐treatment initiation. Sixty‐four percent of individuals achieved efficacy with a mean Phe of 231 μmol/L (60% decrease) after 14 months at mean dose of 37.4 mg/day. Common side effects included injection site reactions in 77%, joint pain in 64%, rash in 45%, headaches in 26%, fatigue in 19%, fever and/or chills in 13%, GI symptoms in 13%, and anaphylaxis in 9%. Discontinuation occurred in 24.5% of this cohort, with rates significantly higher in 12th graders (40%) and college students (32%) versus 9th–11th graders (5.5%). Pegvaliase may lower Phe levels in adolescents, reaching target blood Phe goals (≤ 360 μmol/L) once efficacy is achieved. Treatment showed better sustainability when initiated earlier in adolescence. The higher discontinuation during transitional years (12th grade/college) suggests treatment challenges increase during these periods. Earlier initiation, when family support is typically stronger, may improve outcomes. These findings support reconsidering current age restrictions for pegvaliase therapy.

## Key Points/Highlights

- Adolescents with PKU on pegvaliase achieved a significant reduction in blood Phe, demonstrating the efficacy of pegvaliase in this population overall.
- Average pegvaliase dosing and side effect profiles were similar in this adolescent cohort compared to adults in clinical trials, whereas time to efficacy was slightly longer in this adolescent cohort.
- Younger adolescents showed lower discontinuation rates on pegvaliase possibly due to a stronger support system or decreased stressors that accompany later adolescent transition years.

## 1 Introduction

Phenylketonuria (PKU) is an autosomal recessive genetic disorder caused by an inborn error in phenylalanine (Phe) metabolism due to insufficient activity of the enzyme phenylalanine hydroxylase. A complete or partial deficiency of this enzyme results in elevated levels of Phe in the blood and brain leading to cognitive impairment, abnormal brain imaging findings including white matter changes, as well as heightened risk of mood disorders, anxiety, and challenges in attention and executive functioning [1, 2, 3]. To alleviate these adverse effects and optimize development and neurocognitive function, PKU is diagnosed early in infancy after presumptive positive newborn screening (NBS), in order to initiate Phe‐lowering treatment in the neonatal period. This treatment is continued over the lifespan with the goal of maintaining blood Phe levels ≤ 360 μmol/L [4]. Despite early diagnosis and treatment, there remain suboptimal outcomes in PKU treated with diet alone [5].

Since the initiation of NBS for PKU in 1962, the primary treatment has been a diet to limit Phe intake, supported by a Phe‐restricted medical formula to provide optimal nutrition. Due to the strictness of the protein limitation and high volume of medical formula required, sustainability of the diet is challenging. As children transition into adolescence and adulthood, adhering to the diet becomes increasingly difficult. Adolescents encounter hurdles, including transitioning to greater independence, less parental oversight, heightened social pressures around eating, and added responsibilities, all of which can hinder adherence to a low‐Phe diet [2, 6]. Additionally, the diet becomes more challenging in adolescence due to an increase in caloric requirements for growth without a simultaneous increase in dietary Phe tolerance. The effect of this caloric discrepancy requires the growing adolescent to take in a very high number of low‐Phe calories to sustain growth and maintain blood Phe levels within the target treatment range.

The American College of Medical Genetics and Genomics (ACMG) advises maintaining blood Phe concentrations ≤ 360 μmol/L for all ages [4]. Relaxing control of dietary Phe intake is deleterious and may result in executive function deficits and psychiatric symptoms, including anxiety and depression, which can lead to lower educational achievement and hinder efforts to regain metabolic control [7]. Notably, adherence to these guidelines decreases with age, from 73% of children aged 5–12 maintaining blood Phe within goal, and then falling to 50% in those aged 13–17, 37% in young adults aged 18–29, and 29% in adults 39 years and older [8]. Few approved treatment options exist for adolescents, and those available must typically be used alongside a Phe‐restricted diet in most individuals with PKU.

In older adolescents and adults, large neutral amino acids (LNAA) are sometimes used with the intention of blocking the absorption of Phe from the intestine and across the blood–brain barrier [7]. While theoretically decreasing brain Phe, this therapy does not substantially reduce blood Phe levels [9]. This is used on a limited basis in clinical practice for those with elevated blood Phe levels despite other treatment attempts.

In 2007, sapropterin dihydrochloride (Kuvan, BioMarin Pharmaceutical, Novato, CA) became the first available blood Phe lowering pharmacologic treatment option. Sapropterin dihydrochloride is an analog for BH_4_, the natural cofactor for phenylalanine hydroxylase. However, sapropterin is only effective for a subset of individuals who retain some enzyme activity; therefore, many individuals with classical PKU do not have a meaningful response. Those with PKU who respond to sapropterin may be able to relax their protein restriction but typically will need to continue some level of dietary therapy in order to maintain blood Phe levels within the target treatment range. Individuals who respond to sapropterin therapy may experience improved neurocognition and psychosocial function [7].

In 2018, the US Food and Drug Administration (FDA) approved pegvaliase, an enzyme substitution therapy. Therapeutic response to pegvaliase allows for lower blood Phe levels, increased natural protein intake, and a reduction in reliance on medical formula. Currently, pegvaliase is approved for treating PKU in individuals with blood Phe levels of ≥ 600 μmol/L aged 18 and older in the United States, and for those aged 16 and older in the European Union (EU) [4]. Since its approval, numerous metabolic providers in the United States have begun administering pegvaliase to adolescents, including those under 18 years of age.

Starting pegvaliase therapy requires a considerable commitment of time and logistics, along with awareness of common and possible severe side effects. Pegvaliase consists of a polyethylene glycol‐conjugated enzyme, phenylalanine ammonia lyase (PAL), given via subcutaneous injection, with a standard dosage range of 20–60 mg daily. As a non‐human enzyme, it poses immunogenic risks, potentially leading to the formation of antibodies that could trigger adverse reactions, ranging from minor injection site reactions to severe allergic reactions including anaphylaxis [10].

To minimize immunogenic responses, it is recommended to initiate therapy with a regimen that includes induction, titration, and maintenance phases, with consideration for the use of premedication [10]. Based on drug labeling guidelines, the treatment should start with a 10‐week regimen. This begins with an induction phase where a dose of 2.5 mg is administered once a week for the first 4 weeks. Following this, a titration phase involves gradually increasing the dosage or frequency starting with 2.5 mg twice weekly up to 10 mg daily. The individual is then increased to 20 mg, which is considered reaching the maintenance phase. The dosage may be increased to up to 60 mg daily, based on the current prescribing label, depending on when efficacy is reached [11]. It often takes a few months to over a year to reach the target dose that lowers blood Phe into the treatment range without the use of medical formula or a Phe‐restricted diet.

This paper explores the experience of the treatment of adolescents with pegvaliase therapy from several US clinical sites, aiming to understand factors that maximize the likelihood of treatment success. The World Health Organization (WHO) and medical professionals increasingly use a broad definition of adolescence in describing the shift from childhood to adulthood that can last from ages of 10 to mid‐twenties [12, 13, 14]. For the purpose of this manuscript, we wanted to compare life stages within this broader range of adolescence, and thus collected information about individuals with PKU up to age 22 years, equivalent to late college age. The adolescent period is a critical window for management, both for allowing optimal neurocognitive and psychiatric function and for setting the stage for maintaining PKU treatment in adulthood. Therefore, effective management for adolescents is a crucial step for lifelong PKU treatment success.

## 2 Methods

Data was collected using retrospective chart review from four US metabolic centers for individuals with PKU who began treatment with pegvaliase at age 22 or younger. Data were collected through March 2025. Data collectors complied with site‐specific data sharing requirements and sent de‐identified data to investigators based at Boston Children's Hospital for review and analysis. A spreadsheet was created and distributed across sites to specify the data that was to be collected. The spreadsheet described the endpoints to be collected. The tool specified the units of measure for blood Phe levels as well as the terminology to be used to describe outcomes such as side effects or adverse events (AEs).

Data collected included demographic data, pre‐initiation data, pegvaliase response and blood Phe level data, and side effects or AEs. Specifically, we collected age and sex, comorbidities, grade at start, living situation, and motivation for starting treatment. Pegvaliase response information was collected, including date of first blood Phe response (defined as 50% reduction), pegvaliase dose at first blood Phe response, date of full efficacy (defined as blood Phe ≤ 360 μmol/L with intact protein intake ≥ 40 g, off of sapropterin if applicable), grade level at full efficacy, and pegvaliase dose at full efficacy. Blood Phe level information included mean level prior to pegvaliase start, mean level in the past year at time of data collection, mean level since achieving full efficacy (as applicable), mean level since starting pegvaliase for those without full efficacy, mean level at discontinuation (as applicable), and mean level since discontinuing pegvaliase (as applicable). Side effect information during different phases of treatment titration was collected as well as whether individuals had been prescribed steroids or been hospitalized for side effects. When applicable, discontinuation information was collected, including pegvaliase end date, pegvaliase dose at discontinuation, living situation, grade in school, whether full efficacy was achieved at time of discontinuation, and reason for discontinuation.

All submitted data met eligibility criteria, and no data were excluded from analysis. No analysis for statistical significance was conducted due to the small cohort.

## 3 Results

This study aimed to explore factors associated with pegvaliase treatment success and treatment discontinuation in the adolescent period, encompassing individuals aged 12–22 years at the initiation of treatment. No individuals < 14 years old were identified in the available records.

This report includes data on 53 individuals, comprising 28 high school students, 18 college students, and seven post‐high school individuals not enrolled in college at the start of pegvaliase treatment. Among the participants, there were 30 males and 23 females. The average age was 18.2 years (with a standard deviation of 1.95 years), ranging from 14.5 to 22.9 years (Table 1). Two individuals from one site had started pegvaliase as adolescents as part of the PRISM pegvaliase clinical trial.

**TABLE 1:** Demographics of individuals at the time of first pegvaliase injection.

Grade^a^	Male^b^	Female^b^	Total
9th grade	1	2	3
10th grade	4	3	7
11th grade	6	2	8
12th grade	5	5	10
College—1st year	6	5	11
College—2nd year	2	0	2
College—3rd year	1	2	3
College—4th year	2	0	2
Post High school^c^	3	4	7

Age (years)^d^	Male	Female	Total
14	1	2	3
15	3	0	3
16	5	4	9
17	4	1	5
18	7	10	17
19	4	3	7
20	3	1	4
21	0	2	2
22	3	0	3
Total	30	23	53
Mean Age			18.2

^a^ Grade in school at initiation of pegvaliase treatment (if in between grades, the grade for the semester following the break is indicated).

^b^ Sex assigned at birth.

^c^ Post‐high school encompasses individuals not enrolled in college at the start of pegvaliase treatment.

^d^ Age in years at time of first pegvaliase injection.

Based on the symptom frequency analysis, the most commonly reported comorbidity was anxiety, appearing in 20 individuals (31%). This was followed by depression, documented in 11 individuals (17%), and ADHD reported in 10 individuals (15%). Other frequently observed comorbidities included obesity in six individuals (9%), heart defect in four (6%), and executive dysfunction in four (6%). There were two individuals (4%) that also reported a history of motor tics, eating disorder, intellectual disability, and panic attacks. One individual (2%) had a history of lymphoma in remission. The actual comorbidities may be higher as these were reported by the medical team from record review and not systematically by the individuals themselves.

In provider review of records as indicated in the free response fields, 41/53 individuals had a discernable motivation for starting pegvaliase. Primary motivations included to stop current PKU treatment regimen and eat a regular diet (37/41 responses), to improve quality of life (2/41), to optimize blood Phe control (2/41), which was also implied as a motivation in other free response answers, and to maximize performance in sports (1/41).

For the full adolescent cohort, the mean blood Phe level was 716 μmol/L (range 181–1806) in the year prior to pegvaliase start and was 424 μmol/L (range 7–1418) over the past year at the time of the data collection. Blood Phe levels over the past year were based on 47 out of 53 individuals, as 6 individuals had not submitted levels in over a year. There were 34/53 (64%) individuals who achieved efficacy. The mean blood Phe level over the past year for those who had achieved full efficacy or since achieving full efficacy was 231 μmol/L (range 7–829), based on 33 out of 34 individuals (as 1 individual had not submitted levels in over a year). The mean blood Phe level over the past year for those who had discontinued or since discontinuation was 844 μmol/L (range 341–1418), based on 9 out of 12 individuals.

When comparing the mean blood Phe levels of our adolescents the year prior to pegvaliase start date and the mean blood Phe level since starting treatment, a 44% reduction in levels was observed overall, and a 60% drop was observed in responders. Average time to first response was 6.73 months, with an average dose at first response of 27.6 mg/day (range 2.5–60) based on 35 individuals with data who achieved an initial response. The average time to full efficacy was 14 months. The average pegvaliase dose to reach full efficacy was 37.4 mg/day, based on 32 individuals with available data.

After reaching efficacy, providers used customized dosing regimens with doses based on an individual's clinical status and ranging from 45 mg to 400 mg/week (average 225 mg/week or 32.2 mg/day).

**FIGURE 1:**
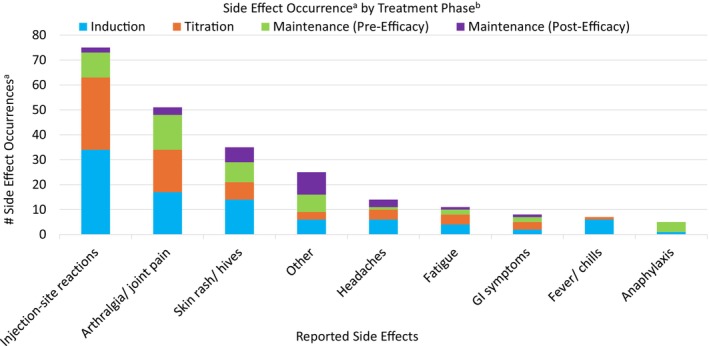
Side effect occurrence by treatment phase. ^a^Occurrence indicates that the individual experienced at least one episode of a specific side effect during a particular phase. ^b^Treatment phases (induction, titration, maintenance [pre‐efficacy], and maintenance [post‐efficacy]) are based on dosing progression.

The most common side effects or AEs noted from most to least frequent were injection site reactions in 41/53 (77%), joint pain/arthralgias in 34/53 (64%), skin rash/hives in 24/53 (45%), headaches in 14/53 (26%), fatigue in 10/53 (19%), fever and/or chills in 7/53 (13%), GI symptoms in 7/53 (13%), and anaphylaxis in 5/53 (9%) (Figure 1). Other various side effects were reported in 20/53 (38%), including dizziness, blurred vision, irritability, cough and congestion, and injection site pain. Side effects were reported most frequently during earlier phases of treatment, with 90 side effect occurrences reported during induction, 68 during titration, 48 during pre‐efficacy maintenance, and 25 during post‐efficacy maintenance dosing. There were no hospitalizations related to pegvaliase, but one individual had reported an emergency department (ED) visit. There was one individual who had anaphylaxis twice, once during induction and once while at a maintenance dose before efficacy, which led to discontinuation. AEs were not included for the two individuals formerly in the PRISM trial, as only clinic information was available for use.

**FIGURE 2:**
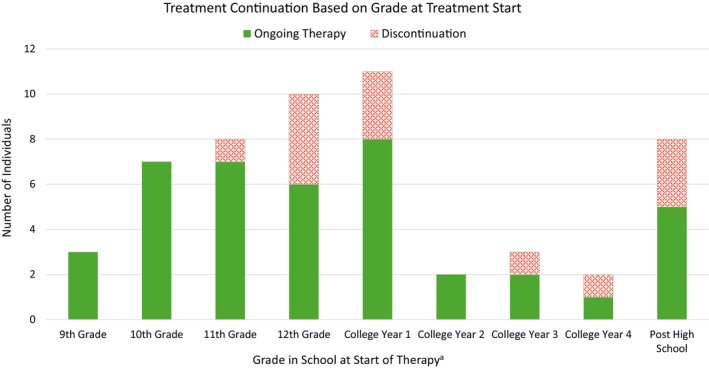
Treatment continuation based on grade at treatment start. ^a^College year based age of individual assuming typical 4 year college track.

Thirteen individuals (24.5%) discontinued treatment, and their average blood Phe level at/before discontinuation was 906 μmol/L (382–1441) (Figure 2). At the time of discontinuation, 4 individuals were in their last year of high school, 5 were in college, and 4 had completed high school but were not in school. Of those who started pegvaliase in high school grades 9–11, only 1/18 (5.5%) discontinued (due to injection fatigue while in college but has since restarted pegvaliase therapy). Of those who started in 12th grade of high school, 4/10 (40%) discontinued, and of those who started while in college or college age, 8/25 (32%) discontinued. All but one of the discontinuations had not yet reached efficacy; notably, the individual who did reach efficacy and discontinued was in 12th grade, and she restarted pegvaliase 4.5 years later. Reasons for discontinuation included side effects (4), lack of efficacy (2), and one report each of issues with insurance coverage, too busy, injection difficulties, injection fatigue, interest in a clinical trial, leaving for college, and difficulty with treatment due to organization and mental health. Under the comments regarding discontinuations, one was late diagnosed, one had poor adherence, and a variety of mental health diagnoses were noted.

## 4 Discussion

The average age of our participants was 18.2 years, compared to the average age of 29.2 years of participants in the PRISM study, which evaluated the safety and efficacy of pegvaliase for the treatment of adults with phenylketonuria. A comparison of the findings from this study to the PRISM study highlights differences and similarities of pegvaliase use in these two different populations.

Prior to initiating treatment, the mean blood Phe level over the past 12 months was 716 μmol/L in this adolescent cohort, compared to the baseline blood Phe level of 1232.7 μmol/L in the PRISM trial, which is expected given that most adolescents were on PKU treatment prior to pegvaliase start but were not able to achieve the level of adherence needed to bring blood Phe levels into target treatment range. After pegvaliase initiation, the mean blood Phe level for this adolescent cohort over the past year was 424 μmol/L, which was a 44% decrease. Among adolescents who had achieved the definition of full efficacy, the mean blood Phe level over the past year or since achieving efficacy was 231 μmol/L (range 7–829), a 60% drop based on 33 out of 34 individuals. This significant blood Phe level decrease demonstrates that pegvaliase is an effective option for many adolescents in lowering blood Phe levels to the goal of ≤ 360 μmol/L. In comparison, the mean blood Phe concentration at 12 months in the PRISM trial population was 565 μmol/L, a 51.1% decrease from baseline, and at 24 months was 311 μmol/L, a 68.7% decrease from baseline [10]. The percentage decline in the adolescent cohort in this study was relatively less, likely due to the lower mean baseline blood Phe level.

Average time to efficacy (defined as blood Phe level ≤ 360 μmol/L with at least 40 g dietary protein and off sapropterin if applicable) in the adolescent cohort was 14 months, slightly longer than the time to sustained blood Phe response in the PRISM trial population, which was 10.7 months [15]. This discrepancy in time to efficacy is likely the result of a slower dose titration in clinical practice compared to what was required in the PRISM clinical trial protocol. Average daily doses for efficacy in the adolescent population appear to be consistent with other studied cohorts [10, 16].

The most reported side effects experienced by this adolescent cohort were similar to the AEs in the adult PRISM trial population. Overall, in both cohorts, the AE/side effect profile appeared to improve with time on treatment. AE rates in the PRISM trial decreased from 58.6 per person‐year in early treatment (less than 6 months) to 19.4 per person‐year in late treatment (more than 6 months). In our adolescent population, reported side effects were more frequent in earlier phases of treatment compared to maintenance stages, similar to the PRISM trial population. Once individuals had achieved efficacy, the side effects were lowest. AEs/side effects were among the reasons for discontinuation in both study populations.

Among adolescents, almost all (93%) of the discontinuations had not yet reached efficacy. Failure to reach efficacy was a reason cited for the decision to discontinue treatment. We observed an overall 24.5% discontinuation rate in our cohort compared to a 33.7% discontinuation rate in the adult PRISM trial population. Notably, only 1 out of 18 adolescents (5.5%) who initiated treatment in 9th to 11th grade of high school discontinued treatment. However, of those who started their senior year of high school, 4 out of 10 (40%) discontinued, and of those who started the first year of college, 3 out of 11 (27%) discontinued. For those who started while in college or at college age, 8 out of 25 (32%) discontinued. The discontinuation rate was much lower for the age range prior to 12th grade.

These trends in discontinuation observed may be related to the challenges associated with the early treatment phase of pegvaliase, including the increased occurrence of AEs/side effects and the stress associated with initiating a new medical treatment into one's lifestyle. This suggests that starting pegvaliase in earlier adolescence may be beneficial given the higher level of familial support versus waiting until later adolescence when there are significant life transitions and increased independence that come with entering college or the workforce. It is possible that adolescents who began treatment during early high school were more likely to successfully manage these challenges, since it is a time when one often has increased parental oversight, support, and less outside responsibility in comparison to the transitional phase of 12th grade to college age.

## 5 Conclusions

Pegvaliase treatment can significantly improve quality of life for those who respond since it can improve blood Phe control and allow diet normalization. However, it is also associated with a significant treatment burden due to requirement for daily injections and a significant side‐effect profile. It is well understood that family support plays a big role in adherence to difficult treatments, including both dietary and medical management for PKU. Many aspects of treatment with pegvaliase are facilitated by support of family members to help with health insurance, ordering medication and formula, organization, injection observation, management of side‐effects, and sometimes even help with injecting.

Time and dose required for efficacy varied greatly based on individual factors and are unpredictable. These findings are overall consistent with what has been previously published on the adult population, and are due at least in part to the immune response to phenylalanine ammonia lyase and pegylation.

Mid‐ to late‐adolescence is a vulnerable period in general with gaining independence and new responsibilities. It is also a time that blood Phe level control may deteriorate in some individuals. This decline in PKU control compounds the difficulties of transitioning to adulthood as it may contribute to executive function deficits, decreased attention, anxiety, and depression. Our cohort's findings included that 12th grade and first year of college were associated with higher discontinuation of pegvaliase, which are adolescent years of the most significant life events and transitions. This higher discontinuation rate was not just constrained to the first year of college but through all college years for those attending and college‐aged young adults not attending college. During these stages of life, the older adolescent is assuming independence for many aspects of their lives. Adding the burden of a new treatment that requires significant commitment, invariable physical side effects, and organization/executive function demands also puts these individuals at higher risk for treatment failure.

Conversely, there was unusually high treatment success in those who started pegvaliase in 9th to 11th grade, with only one later discontinuing treatment while in college (and has since resumed pegvaliase), and with most having achieved full efficacy. The four centers that contributed data have large, well‐established PKU programs and providers that are experienced with pegvaliase in clinical care and clinical trials. This could have contributed to the lower discontinuation rates in the younger individuals. The adolescents in this study younger than 18 years had also been approved for off‐label use by their clinics, likely because they had qualities determined to be helpful to their success, including a high level of familial support. Future efforts may include determining more precisely the logistical and environmental factors that would set up individuals within this age group for treatment success and continuation of this therapy.

In conclusion, we propose that starting pegvaliase at an age younger than 18 years in order to allow adolescents to have a more supportive environment and have a higher likelihood for treatment success.

## Author Contributions

**Suzanne Hollander:** (guarantor author) conception, design, acquisition of data, analysis of data, interpretation of data, drafting (original), reviewing, oversight. **Briana Valli:** conception, design, acquisition of data, analysis of data, interpretation of data, drafting (original), reviewing. **Erika Vucko:** acquisition of data, reviewing. **Melissa Lah:** acquisition of data, reviewing. **Amarilis Sanchez‐Valle:** acquisition of data, reviewing. **Brittany M. Murray:** acquisition of data, design, reviewing. **Amy Kritzer:** acquisition of data, design, reviewing. **Stephanie Sacharow:** conception, design, acquisition of data, analysis of data, interpretation of data, drafting (original), reviewing, oversight.

## Funding

The authors have nothing to report.

## Ethics Statement

Each site gathered data under regulations of their institutional requirements.

## Conflicts of Interest

Suzanne Hollander has received financial contributions for speaking engagements or consulting from PTC Therapeutics, Vitaflo NA, Biomarin Pharmaceuticals and Sanofi. Erika Vucko has received financial contributions for speaking engagements or consulting and/or research funding from BioMarin Pharmaceuticals, Maze Therapeutics, PTC Therapeutics and and Otsuka. Melissa Lah has received financial contributions for speaking engagements and/or research funding from PTC Therapeutics, ModernaTx, Synlogic Operating Company Inc, Homology Medicines Inc, and BioMarin Pharmaceutical Inc. Amarilis Sanchez‐Valle has received financial contributions for speaking engagements and research funding from Biomarin Pharmaceuticals. Brittany M. Murray has received financial contributions for speaking engagements or consulting from Biomarin Pharmaceuticals and PTC Therapeutics. Stephanie Sacharow has received financial contributions for speaking engagements or consulting and/or research funding from Jnana, Biomarin Pharmaceuticals, PTC Therapeutics, and Synlogic.

## Data Availability

Data were collected under regulations of each institutional site from their electronic medical records and aggregated in spreadsheets using protected servers. Research data are not shared.
